# Development of NASH in Obese Mice is Confounded by Adipose Tissue Increase in Inflammatory NOV and Oxidative Stress

**DOI:** 10.1155/2018/3484107

**Published:** 2018-07-02

**Authors:** David Sacerdoti, Shailendra P. Singh, Joseph Schragenheim, Lars Bellner, Luca Vanella, Marco Raffaele, Aliza Meissner, Ilana Grant, Gaia Favero, Rita Rezzani, Luigi F. Rodella, David Bamshad, Edward Lebovics, Nader G. Abraham

**Affiliations:** ^1^Departments of Medicine, Pharmacology and Gastroenterology, New York Medical College, Valhalla, NY 10595, USA; ^2^Department of Medicine, University of Padova, Padova, Italy; ^3^Department of Drug Science, University of Catania, Catania, Italy; ^4^Anatomy and Physiopathology Division, Department of Clinical and Experimental Sciences, University of Brescia, Brescia, Italy; ^5^Marshall University, Joan C. Edwards School of Medicine, Huntington, WV 25701, USA

## Abstract

**Aim:**

Nonalcoholic steatohepatitis (NASH) is the consequence of insulin resistance, fatty acid accumulation, oxidative stress, and lipotoxicity. We hypothesize that an increase in the inflammatory adipokine NOV decreases antioxidant Heme Oxygenase 1 (HO-1) levels in adipose and hepatic tissue, resulting in the development of NASH in obese mice.

**Methods:**

Mice were fed a high fat diet (HFD) and obese animals were administered an HO-1 inducer with or without an inhibitor of HO activity to examine levels of adipose-derived NOV and possible links between increased synthesis of inflammatory adipokines and hepatic pathology.

**Results:**

NASH mice displayed decreased HO-1 levels and HO activity, increased levels of hepatic heme, NOV, MMP2, hepcidin, and increased NAS scores and hepatic fibrosis. Increased HO-1 levels are associated with a decrease in NOV, improved hepatic NAS score, ameliorated fibrosis, and increases in mitochondrial integrity and insulin receptor phosphorylation. Adipose tissue function is disrupted in obesity as evidenced by an increase in proinflammatory molecules such as NOV and a decrease in adiponectin. Importantly, increased HO-1 levels are associated with a decrease of NOV, increased adiponectin levels, and increased levels of thermogenic and mitochondrial signaling associated genes in adipose tissue.

**Conclusions:**

These results suggest that the metabolic abnormalities in NASH are driven by decreased levels of hepatic HO-1 that is associated with an increase in the adipose-derived proinflammatory adipokine NOV in our obese mouse model of NASH. Concurrently, induction of HO-1 provides protection against insulin resistance as seen by increased insulin receptor phosphorylation. Pharmacological increases in HO-1 associated with decreases in NOV may offer a potential therapeutic approach in preventing fibrosis, mitochondrial dysfunction, and the development of NASH.

## 1. Introduction

Metabolic syndrome and its associated pathologies of obesity, insulin resistance (IR), and dyslipidemia are often accompanied by liver involvement, defined as nonalcoholic fatty liver disease (NAFLD) [1]. NAFLD's progression to nonalcoholic steatohepatitis (NASH), characterized by low grade inflammation, cell ballooning, and mitochondrial dysfunction, is a primary risk factor for development of fibrosis and cirrhosis and therefore an important area of clinical research [2]. Chronic, low grade inflammation due to metabolic syndrome is provoked when the capacity for adipocytes to store fat is overwhelmed resulting in the production of inflammatory cytokines leading to metabolic inflammation [3]. Fatty acids released from hypertrophic, dysfunctional, and insulin resistant adipocytes, together with increased hepatic de novo lipogenesis and impaired (FA) fatty acid export, cause an accumulation of triglycerides in the liver leading to lipotoxicity [4]. Increased calorie intake and obesity lead to an increase in tissue fat mass through adipocyte hyperplasia and hypertrophy, subsequently resulting in a decrease in adiponectin and an increase of inflammatory TNF-*α* causing IR, inflammation, and oxidative stress in the liver [5]. Reactive oxygen species (ROS) can induce lipid peroxidation leading to inflammation and liver damage [6, 7]. Steatotic livers are more sensitive to increased ROS and oxidative stress, leading to mitochondrial dysfunction, decreased levels of hepatocyte antioxidants, and inflammation, and culminating in NASH and fibrosis [8, 9].

Induction of HO-1, an antioxidant gene highly inducible in a range of cells, including erythrocyte phagocytosing Kupffer cells and splenic macrophages and in all organs, including fat tissues, but excluding testes and brain, confers advantageous effects in metabolic syndrome [10, 11]. Cobalt protoporphyrin (CoPP) induction of HO-1 contributes to the phosphorylation of the insulin receptor, thus improving insulin sensitivity [12, 13]. Additionally, HO-1 acts through the degradation products of the prooxidant heme, bilirubin, and biliverdin, antioxidants that increase mitochondrial fusion, while also serving to improve adipocyte function and remodeling by increasing levels of adiponectin expression [14, 15]. Humans with low levels of HO-1 suffer severe oxidative stress and organ failure and demonstrate iron deposits in the liver [16, 17]. CoPP has been used to prevent body weight gain, increase oxygen consumption, and decrease fasting blood glucose in rats and mice [18] (reviewed in [11]). Recently, CoPP decreased expression of proapoptotic protein, abridge percolation of inflammatory cells, and reduced AST and ALT levels in IR induced liver damage [19, 20]. Moreover, HO-1 induction increased mitofusion over fission related proteins and improved mitochondrial quality control [21].

In adipose tissues, induction of HO-1 has been shown to reduce body weight, decrease NOV, and increase PGC-1*α* mediated thermogenesis, thereby increasing energy uptake and the stimulation of mitochondrial FA oxidation [22]. Adipose PGC-1*α* serves as a key moderator of energy metabolism and promotes mesenchymal stem cell differentiation into brown fat adipocytes [23] and the browning of white fat to a distinct phenotype known as brite fat, which aids in the prevention of the development of metabolic syndrome and type 2 diabetes mellitus (T2DM) [24].

Another important component of metabolic syndrome is an increase in the levels of the prooxidant heme. Intracellular heme levels play a central role in the regulation of many cell functions [25–27]. Inflammatory increases in IL-6 upregulate hepcidin and iron trapping in Kupffer cell diseases [1]. The resulting increase in cellular heme decreases levels of PGC-1*α*, lipid metabolism, and adipogenesis [28].

The recently discovered inflammatory adipokine, NOV/CCN3 gene (nephroblastoma overexpressed) [29], is also shown to be repressed in conditions of increased HO-1 levels [22]. This protein plays key roles in inflammation, wound healing, fibrosis, and cancers [29]. NOV is involved in the adhesion, migration, proliferation, differentiation, and survival of different cell types [29] and modulates the expression of inflammatory molecules [30, 31].

In adipose tissues, induction of HO-1 has been shown to reduce body weight, decrease NOV, and increase PGC-1*α* mediated thermogenesis, thereby increasing energy uptake and the stimulation of mitochondrial FA oxidation [22]. Adipose PGC-1*α* serves as a key moderator of energy metabolism and promotes mesenchymal stem cell differentiation into brown fat adipocytes [23] and the browning of white fat to a distinct phenotype known as brite fat, which aids in the prevention of the development of metabolic syndrome and type 2 diabetes mellitus (T2DM) [24].

A recent study in mice has shown that myeloid-specific depletion of NOV exacerbates liver injury in a mouse model of NAFLD [32]. However, global NOV^−/−^ mice fed a HFD have less steatosis compared to WT mice, and hepatic triacylglycerol content is reduced by approximately threefold [29].

We hypothesize that the development of NASH is the result of the combination of increased NOV and inflammation and a decrease of HO-1 in adipose and hepatic tissues leading to the impairment of mitochondrial function. We further propose that induction of HO-1 in adipose tissues will have positive impact on hepatic tissue and reverse the negative effects on NOV levels, decreasing NASH scores and increasing mitochondrial integrity and function.

## 2. Methods

Eight-week-old C57Bl6 male mice were fed a high fat diet (HFD) for 20 weeks, a time frame in which the manifestations of NASH are present. Mice were divided into four groups of 6 animals each: (1) control normal chow diet; (2) HFD; (3) HFD treated for the last 8 weeks with cobalt protoporphyrin (CoPP) (once/week a dose of 5 mg/100 g, bw); and (4) HFD treated for the last 8 weeks with CoPP and the last 3 weeks with tin mesoporphyrin (SnMP at a dose of 20 mg/100 g BW), an inhibitor of HO activity (twice/week). BW at the end of the 20-week period C57 lean, range; 29-31; HF; 56-59, HF-CoPP; 34-39, CoPP-SnMP; 47-51 gm, blood glucose and alanine aminotransferase (ALT) were measured as described [22, 33]. All animal experiments followed the NYMC IACUC institutionally approved protocol in accordance with the NIH guidelines.

### 2.1. Histopathological Examination of Hepatic Tissue, NAS Score Evaluation, and Hepatic Lipid Droplet Analysis

Liver samples from each experimental group were fixed in 4% paraformaldehyde, dehydrated, embedded in paraffin wax, and sectioned (6 *μ*m thick). The main liver histopathological features commonly described in NAFLD including steatosis, inflammation, hepatocyte ballooning, and fibrosis were scored according to the NAFLD histologic activity score (NAS) system, and lipid droplet analysis was performed as previously described [33–35]. Briefly, double-blinded analysis identified the degree of steatosis and NASH (grade 0 ≤ 5%; 1 = 5–33%; grade 2 = 34%–66%; grade 3 ≥ 66%), lobular inflammation (0: no foci, 1 < 2 foci per 200x field, 2: 2 to 4 foci per 200x field, and 3: >4 foci per 200x field), hepatocyte ballooning (0: none; 1: rare or few; 2: many), and fibrosis (0: no fibrosis, 1: perisinusoidal or periportal fibrosis, 2: perisinusoidal and portal/perioral fibrosis, 3: bridging fibrosis, and 4: cirrhosis) [34].

### 2.2. Real-Time qPCR, Western Blot Analysis, HO Activity, Heme Measurement, and O_2_ Consumption

Total RNA and protein were extracted from liver and visceral adipose tissue and gene expression analysis was performed by RNeasy® Lipid Tissue (Qiagen), as indicated by the manufacturer [24, 33]. RNA was determined by measuring the absorbance at 260 nm (A260) with a Biotek™ plate reader and the Take3™ plate (Biotek, Winooski, VT) and assessed by the A260/A280 ratio. cDNA was synthesized from total RNA (Applied Biosystems) using a High Capacity cDNA Reverse Transcription Kit (Applied Biosystems). Real-time PCR was performed using TaqMan® Fast Universal Master Mix (2x), on a 7500 HT Fast Real-Time PCR System (Applied Biosystems). For western blotting analyses, tissue was lysed in RIPA lysis buffer supplemented with protease and phosphatase inhibitors (Complete™ Mini and PhosSTOP™, Roche Diagnostics, Indianapolis, IN as previously described [35]. Heme levels were determined by ELISA (BioVision, Inc., Milpitas, CA) as described [35].

HO activity was determined as described [36]. Briefly, freshly lysed and homogenized tissue samples were incubated in sealed vials at 37°C for 90 min with 30 *μ*M heme, and 2 mM NADPH, in the absence or presence of an HO inhibitor, SnMP (50 *μ*M). After termination of reaction, headspace gas was analyzed for CO with C^13^O^16^ added as an internal standard. CO measurements were performed using an Agilent 5890 GC-MS. HO activity is calculated by subtracting CO levels obtained in the presence of SnMP from those obtained without. Data are normalized to total protein and are presented as pmol CO/mg protein/h.

Mouse oxygen consumption was assessed as described [22, 24]. Briefly, oxygen consumption (VO_2_) and carbon dioxide production (VCO_2_) were measured using the Oxylet gas analyzer and air flow unit (Oxylet; Panlab-Bioseb, Vitrolles, France). Hourly respiratory quotients were measured and performed twice, on individual mice using the VCO_2_ and VO_2_ obtained by the gas analyzer. The results are expressed as the consumed VO_2_ per kilogram body weight per minute (ml/kg/min). The respiratory quota is expressed as CO_2_ eliminated/O_2_ consumed.

### 2.3. Isolation and Development of NOV Overexpressing Adipocytes

Viral transduction was performed as previously described [35]. Briefly, 1x10^6^ cells were seeded per well of a 6-well plate 24 h prior to transduction. The cells were incubated with the transduction medium (1x10^6^ transducing units (TU) of lentiviral particles (Precision LentiORF for NOV (Dharmacon, Lafayette, CO)) in 0.5ml *α*-MEM growth medium supplemented with polybrene (8 *μ*g/ml) for 3h to maximize the contact between each cell and lentiviral particles. Additional culture medium supplemented with polybrene was then added to each well. After an additional 48 h incubation, antibiotic selection medium was used to kill all the nontransduced cells.

### 2.4. Statistical Analyses

Statistical significance between experimental groups was determined by Student's t-test for pairwise comparison between groups or by ANOVA with Tukey-Kramer post hoc analysis for comparison between multiple groups. The data are presented as means ± SEM and the null hypothesis was rejected at p<0.05.

## 3. Results

### 3.1. HO-1 Induction Prevents Fibrosis and Decreases NASH Score

**Figure 1:**
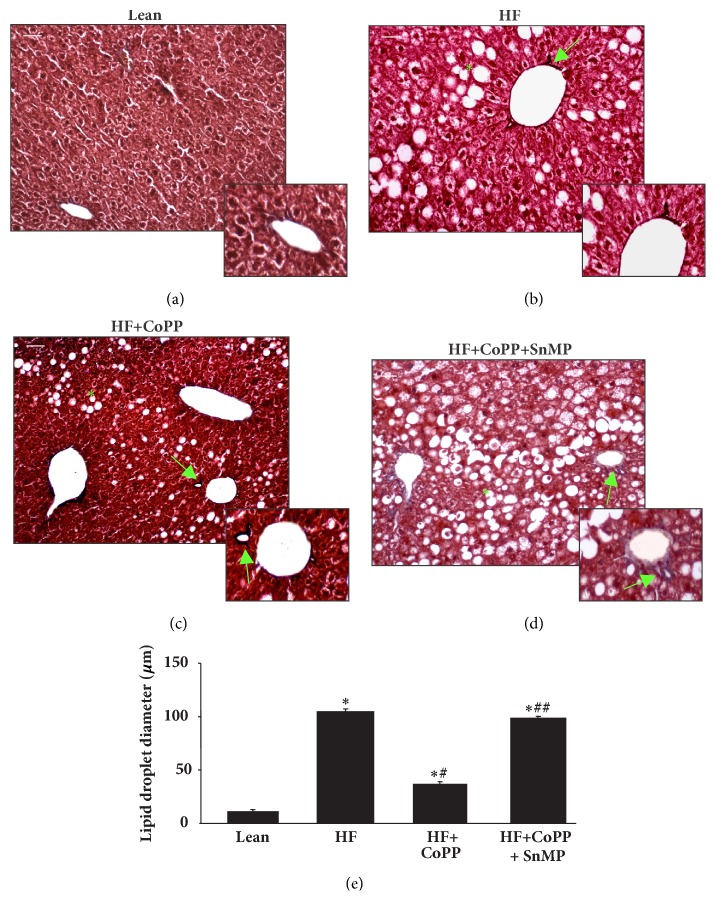
HO-1 induction prevents fibrosis and decreases NASH score. Liver of lean (a), HF fed (b), HF fed treated with CoPP (c), and HF fed treated with CoPP and SnMP (d) in mice and (e) graph summarizes the morphometrical analysis of liver lipid droplet diameter. *∗* p< 0.05 versus lean; # p< 0.05 versus HF fed; and + p< 0.05 versus HF fed mice + CoPP. Masson's trichrome staining. Bar 20 *μ*m. The arrow shows hepatic perivascular fibrosis and (*∗*) indicates the steatosis of control lean mice, HF fed mice, HF fed mice + CoPP, and HF fed mice + CoPP + SnMP.

As expected, livers of lean mice showed no significant evidence of steatosis (0.67% of cells positive for intracellular lipid accumulation) with only rare ballooning and no inflammatory foci and no fibrosis. The livers from HF mice revealed a higher NAS score (NAS: 9) with elevated steatosis, moderate lobular inflammatory loci, significant hepatocyte ballooning, and fibrosis (Table 1; Figures 1(a)–1(e)). Increased HO-1 expression with CoPP improved this score (NAS: 3), diminished all the pathological parameters, and resulted in mild steatosis, rare inflammatory loci and ballooning, and no fibrosis. Inhibition of HO activity in HF mice caused perisinusoidal steatosis and ballooning and portal fibrosis (NAS: 8). Furthermore, the adverse effect of hepatosteatosis was confirmed by detailed morphometrical analysis of liver lipid droplet diameter (Figures 1(a)–1(e)). From these results, we conclude that increased levels of HO-1 can prevent lipid droplet formation in the liver, ultimately preventing the development of NAFLD and NASH in obese mice.

### 3.2. Induction of HO-1 Decreases NOV and Fibrotic Markers and Improves ALT and AST

**Figure 2:**
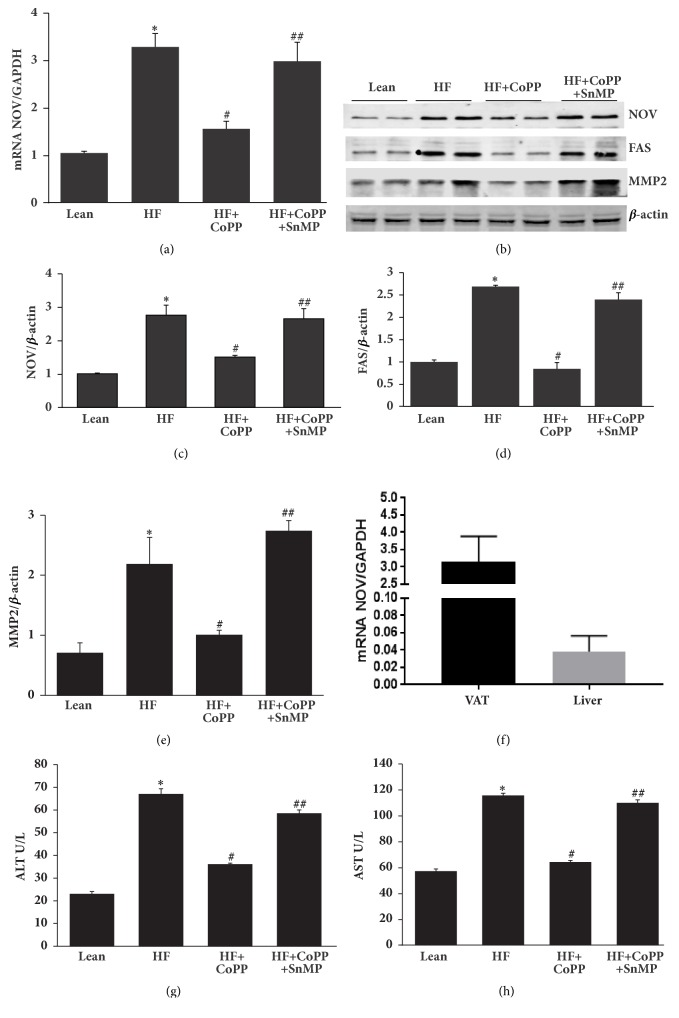
Induction of HO-1 decreases NOV and fibrotic markers and improves ALT and AST. (a) The mRNA expression of NOV, (b) representative western blots and densitometry analysis of (c) NOV, (d) FAS, and (e) MMP2 and levels of (f) AST(U/L), and (g) ALT(U/L) in hepatic tissues of control lean mice, HF fed mice, HF fed + with CoPP, and HF fed mice + CoPP + SnMP. (h) NOV mRNA levels in visceral adipose tissue (VAT) and liver of lean mice. Results are mean ± SE, n=6, *∗*p<0.05 versus lean mice, #p<0.05 versus HF fed mice, and ##p<0.05 versus HF fed mice + CoPP.

As seen in Figure 2(f), levels of NOV in the lean mouse are significantly (p<0.05) higher in visceral adipose tissue (VAT) than in liver tissue. The HF diet increased the expression of hepatic NOV/ CCN3 mRNA and protein content (Figures 2(a), 2(b), and 2(c)) as compared to lean mice (p<0.05). An increase in HO-1 expression resulted in a normalization of NOV expression, an effect that was blocked by an inhibitor of HO activity; SnMP (p<0.05) (Figures 2(a), 2(b), and 2(c)) (p<0.05). Similarly, FAS protein expression was significantly (p<0.05) elevated in HF fed mice and normalized by CoPP (Figures 2(b) and 2(d)). Fibrotic protein signaling in hepatic tissue of obese mice as measured by the expression of MMP2 was reduced by increased HO-1 levels (p<0.05), an effect that was prevented by inhibition of HO activity (Figures 2(b) and 2(e)). Obese mice developed impaired liver function as indicated by increased levels of serum AST (p < 0.05) and ALT (p < 0.05), all of which were normalized by HO-1 induction (p<0.05) (Figures 2(g) and 2(h)). HO-1 reduction in AST and ALT levels was eliminated by SnMP (Figures 2(g) and 2(h)). Taking all of these findings together, it can be concluded that a decrease in levels of the proinflammatory adipokine NOV in conjunction with increased levels of HO-1 mitigates the development of fibrotic markers that contribute to the NASH phenotype.

### 3.3. Induction of HO-1 Decreases Heme and Hepcidin Expression

**Figure 3:**
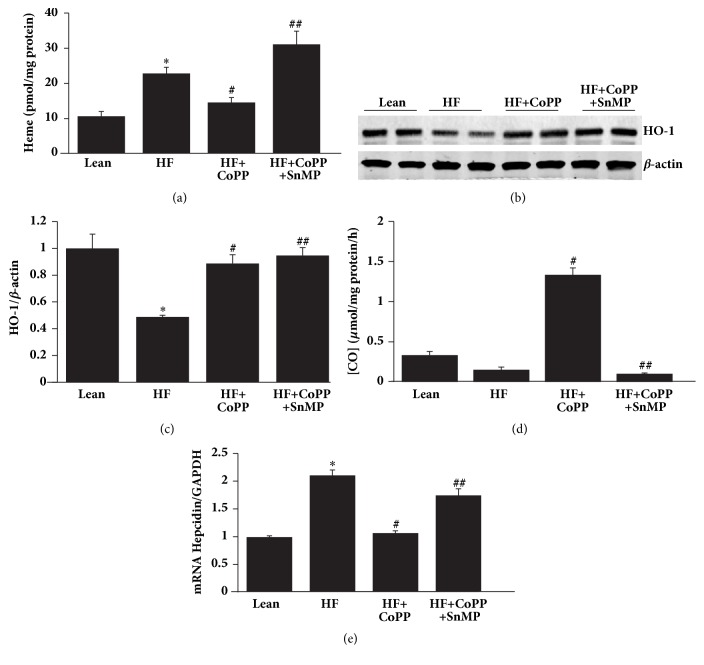
Increase of HO-1 decreases heme levels and hepcidin expression. (a) Heme (pmol/mg protein) content, (b) representative western blots of HO-1, (c) densitometry analysis of HO-1 and (d) CO production (*μ*mol/mg protein/h), and (e) mRNA expression of hepcidin in hepatic tissues of control lean mice, HF fed mice, HF fed mice + CoPP, and HF fed mice + CoPP + SnMP. Results are mean ± SE, n=6, *∗*p<0.05 versus lean mice, #p<0.05 versus HF fed mice, and ##p<0.05 versus HF fed mice treated with CoPP.

In accordance with our hypothesis, our results indicate that NASH livers have significantly (p<0.01) increased heme levels as compared to control lean mice fed a normal chow diet. Induction of HO-1 decreased heme levels as compared to the HF diet group (p<0.01). The favorable effects of induction of HO-1 were reversed by SnMP (Figure 3(a)). Western blot analysis demonstrated that hepatic tissues of mice fed a HF diet for 20 weeks had significantly (P<0.05) decreased levels of HO-1 protein as compared to hepatic tissues of mice fed a normal chow diet (Figures 3(b) and 3(c)). CoPP treatment for 8 weeks increased liver levels of HO-1 as compared to mice fed a HF diet alone, p<0.01 (Figures 3(b) and 3(c)). The positive effects of increased HO-1 levels were reversed by SnMP (Figures 3(b)–3(d)). Of note is the fact that SnMP does not prevent an increase in HO-1 protein expression but rather inhibits HO activity [37]. HO activity in hepatic tissue was increased by CoPP and decreased by SnMP in HF fed mice, p<0.05 (Figure 3(d)). As seen in Figure 3(e), hepcidin mRNA level was increased in NASH livers of HF fed mice, as compared to lean mice. Increased HO-1 expression significantly, p<0.05, reduced the expression of hepcidin, an effect which was reversed by SnMP (p<0.05).

### 3.4. Increase of HO-1 Expression Augments Mitochondrial Integrity

**Figure 4:**
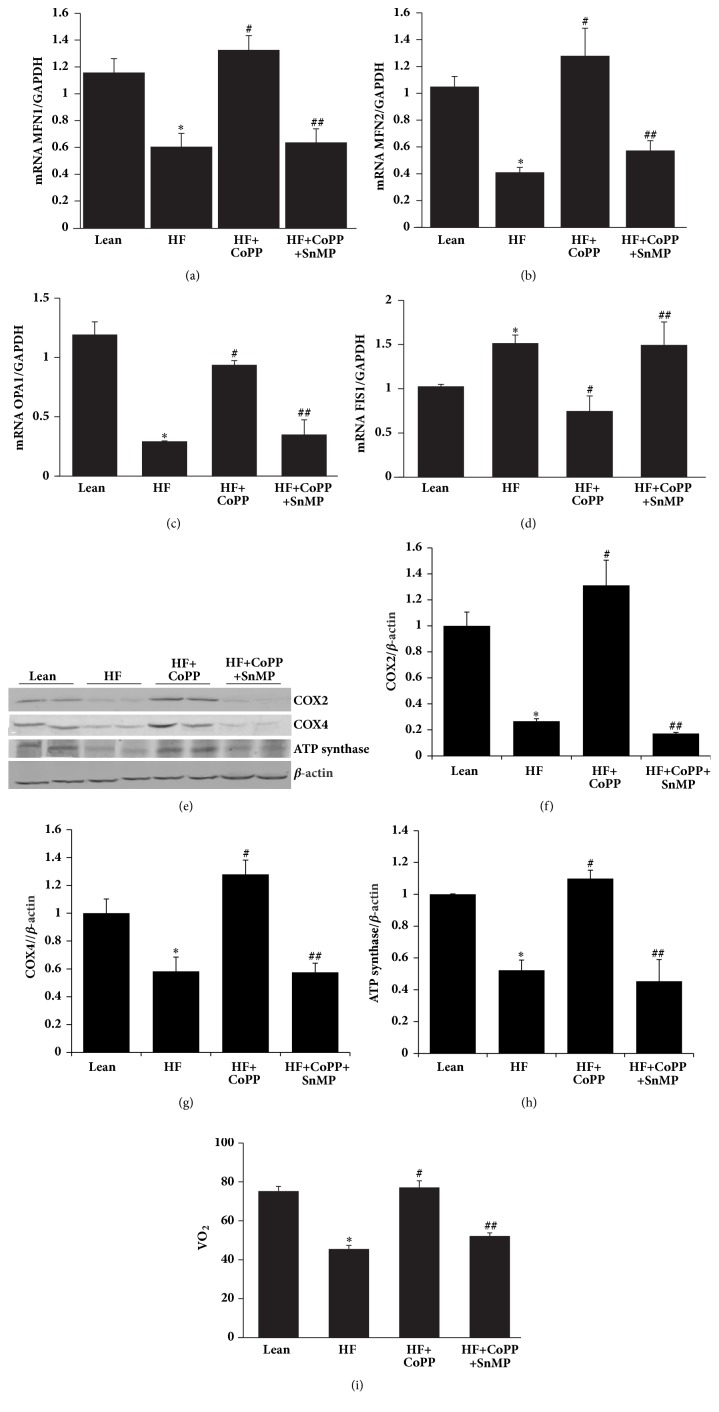
Increase of HO-1 expression augments mitochondrial integrity. mRNA expression of (a) MFN1, (b) MFN2, (c) OPA1, and (d) FIS1. (e) Representative western blots and densitometric analysis of (f) COX2, (g) COX4, and (h) ATP synthase. (i) Total body oxygen consumption (VO_2_) of mice, in hepatic tissues of control lean mice, HF fed mice, HF fed mice + CoPP, and HF fed mice + CoPP + SnMP. Results are mean ± SE, n=6, *∗*p<0.05 versus lean mice, #p<0.05 versus HF fed mice, and ##p<0.05 versus HF fed mice + CoPP.

MFN1, MFN2, and OPA1 expression levels were increased, while FIS1 mRNA was decreased by HO-1 induction (p <0.05) an effect that was reversed by SnMP (p<0.05) (Figures 4(a)–4(d)). These results indicate that HO-1 is a powerful inducer of mitochondrial fusion (the merge of dysfunctional to functional) and an inhibitor of mitochondrial fission. This increase in fusion (MFN1 and MFN2) and decrease in fission (FIS1) contribute to an increase in overall mitochondrial function, leading to a decrease in adiposity in HF fed mice, a consequent reduction in obesity, and a concurrent reduction in the development of NASH. Mitochondrial expression of COX2 and COX4 as well as ATP synthase were reduced in obese mice as compared to lean mice (p<0.05), effects that were reversed by increased levels of HO-1 (p<0.05) (Figures 4(e)–4(h)). Oxygen consumption in obese mice was decreased as compared to lean mice (p<0.05) (Figure 4(i)). However, CoPP treatment of obese mice normalized O_2_ consumption (p<0.05), an effect blocked by SnMP (Figure 4(i)).

### 3.5. HO-1 Upregulation Increases Phosphorylation of the Insulin Receptor in the Liver of Obese Mice

**Figure 5:**
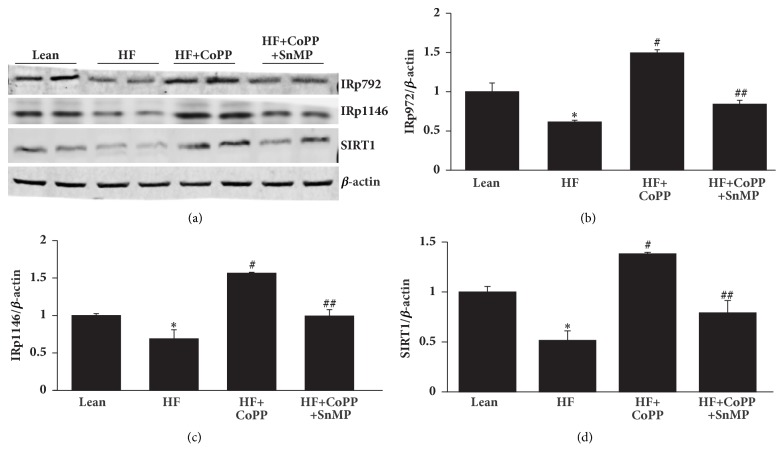
HO-1 upregulation increases hepatic pAKT, pAMPK, and insulin receptor phosphorylation levels. Representative western blots (a) and densitometry analysis of (b) IRp972, (c) IRp1146, and (d) SIRT1 in hepatic tissues of control lean mice, HF fed mice, HF fed mice + CoPP, and HF fed mice + CoPP + SnMP. Results are mean ± SE, n=4, *∗*p<0.05 versus lean mice, #p<0.05 versus HF fed mice, and ##p<0.05 versus HF fed mice + CoPP.

Obese mice had decreased expression levels of IRp-Tyr 972 and IRp-Tyr 1146 (p<0.05), as well as levels of SIRT1, as compared to control lean mice. HO-1 induction ameliorated the effect of HFD on insulin receptor phosphorylation and significantly increased IRp-Tyr 972 and IRp-Tyr 1146, as well as SIRT1 levels (p<0.05) (Figures 5(a)–5(d)). These effects were reversed by SnMP (Figures 5(a)–5(d)). The reversal of these beneficial effects corroborates the role of HO-1 expression and HO activity in mediating the beneficial effects of CoPP.

### 3.6. Adipose Tissue HO-1 Upregulation Increases Phosphorylation of Both the Insulin Receptor and Acetyl-CoA-Carboxylase (ACC)

**Figure 6:**
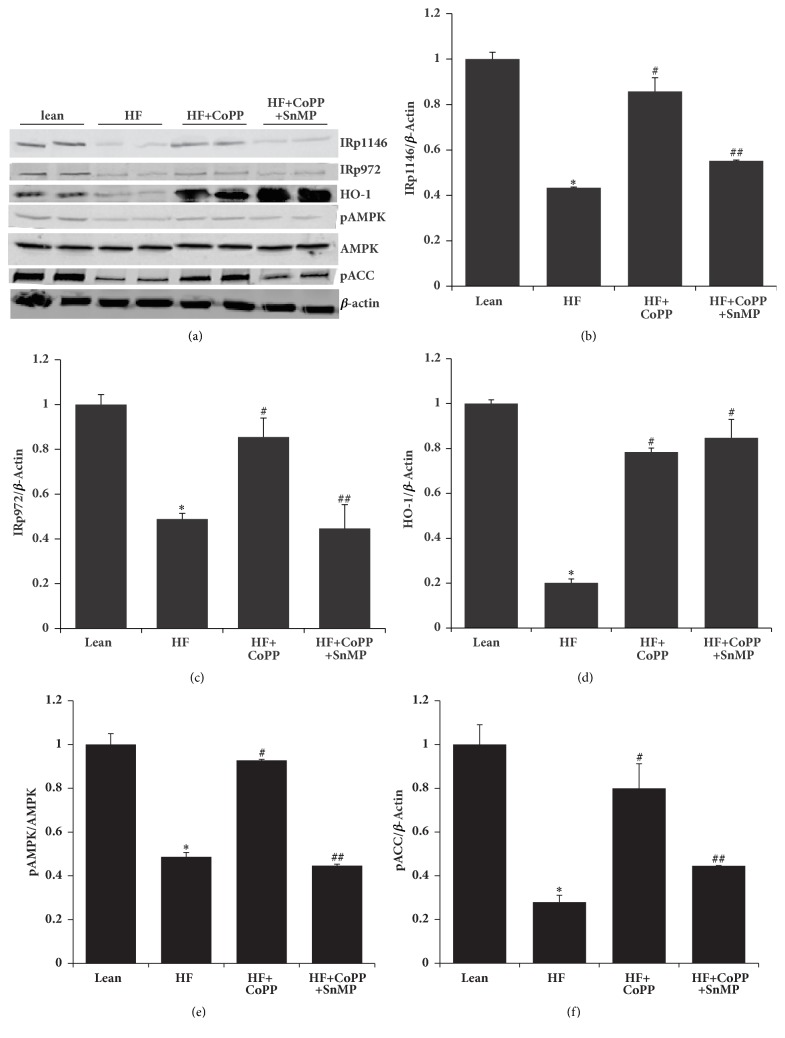
Adipose tissue HO-1 upregulation increases phosphorylation of both insulin receptor and ACC. Representative western blot (a) and densitometry analysis of (b) IRp1146, (c) IRp972, (d) HO-1, (e) pAMPK, and (f) pACC. Results are mean ± SE, n=4, *∗*p<0.05 versus lean mice, #p<0.05 versus HF fed mice, and ##p<0.05 versus HF fed mice + CoPP.

To test the possibility that NOV-mediated increases in adipose inflammation in turn decreases insulin receptor phosphorylation in an obese mouse model, we examined the effects of HO-1 on insulin receptor phosphorylation in adipose tissue. Adipose tissue of obese mice had decreased phosphorylation levels of IRp-Tyr 1146, IRp-Tyr 972, AMPK, and ACC and reduced expression of HO-1 as compared to control lean mice (p<0.05) (Figures 6(a)–6(f)). The negative effects associated with obesity were normalized in obese mice following induction of HO-1 (p<0.05) (Figures 6(a)–6(f)) and reversed by inhibition of HO activity (p<0.05) (Figures 6(a)–6(f)).

### 3.7. Adipose Tissue HO-1 Upregulation Increases Anti-Inflammatory Adiponectin and Mitochondrial Fusion-Associated Proteins, While Decreasing Proinflammatory NOV and the Mitochondrial Fission-Associated Protein, FIS1

**Figure 7:**
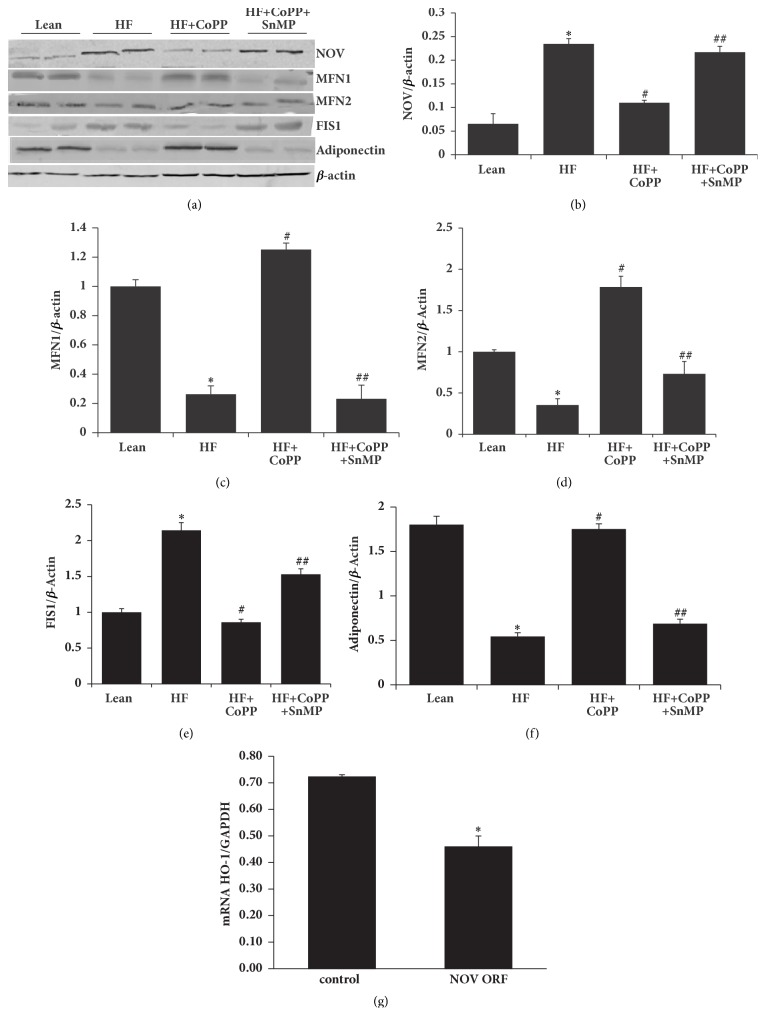
Adipose tissue HO-1 upregulation increases anti-inflammatory adiponectin and mitochondrial fusion-associated proteins, while decreasing proinflammatory NOV and the mitochondrial fission-associated protein, FIS1. (a) Representative western blots, and densitometry analysis of (b) NOV, (c) MFN1, (d) MFN2, (e) FIS1, and (f) adiponectin. (g) HO-1 mRNA levels in NOV overexpressing 3T3-L1 derived adipocytes. Results are mean ± SE, n=4, *∗*p<0.05 versus lean mice/control, #p<0.05 versus HF fed mice, and ##p<0.05 versus HF fed mice + CoPP.

To further test the possibility of potential crosstalk between adipose and hepatic tissue we examined the effects of HO-1 on adipose mitochondrial function as it is related to the proinflammatory adipokine NOV. Inflamed adipose tissue from untreated obese mice expressed elevated levels of NOV. As seen in Figures 7(a) and 7(b), the NOV level in visceral adipose tissues of HF diet fed mice was elevated as compared to the levels in lean mice (p<0.05). HO-1 induction decreased visceral adipose tissue NOV levels (p<0.05), suggesting that induction of HO-1 reprograms white adipose tissue to beige, resulting in less inflammation (Figures 7(a) and 7(b)).

More importantly, as mitochondrial integrity in liver tissue of HF diet fed mice was increased by HO-1 induction and as mitochondrial function is very important also for the health of adipose tissue, we assessed the levels of mitochondrial fusion and fission proteins in the visceral adipose tissue. As seen in Figure 7(a), 7(c)–7(e), the levels of MFN1 and MFN2 were decreased, while FIS1 was increased in obese mice as compared to lean mice, p <0.05. HO-1 induction normalized these levels, an effect reversed by concomitant SnMP-treatment (p<0.05) (Figure 7(a), 7(c)–7(e)). Healthy adipocytes express the anti-inflammatory adipokine, adiponectin. As seen in Figures 7(a) and 7(f), the adiponectin levels in visceral adipose tissues of HF diet fed mice were decreased as compared to the levels in lean mice (p<0.05). CoPP-mediated HO-1 induction normalized visceral adipose tissue adiponectin levels, an effect that was prevented by SnMP-treatment (p<0.05) (Figures 7(a) and 7(f)).

As induction of HO-1 in adipocytes decreased NOV levels, we wondered whether overexpression of NOV would cause a decrease of HO-1 levels. As seen in Figure 7(g), overexpression of NOV in cultured adipocytes led to a reduction in the HO-1 mRNA levels (p<0.05). Successful transduction of cells with the NOV ORF lentiviral particles was confirmed by measuring mRNA levels of NOV in transduced and control adipocytes. The NOV mRNA level was upregulated more than 100-fold (p<0.05) in the NOV overexpressing cells as compared to control cells (data not shown).

## 4. Discussion

The primary findings of this study are the following. (1) The increase of the proinflammatory adipokine NOV and decrease of HO-1 in hepatic and adipose tissue of obese mice is associated with mitochondrial dysfunction and the development and progression of obesity-induced NASH. (2) Fat expansion is associated with remodeling marked by an increase in proinflammatory molecules and oxidative stress and a decrease in PGC-1*α* and insulin receptor phosphorylation with the eventual development of metabolic abnormalities. (3) TNF-*α* and NOV are increased while adiponectin is decreased in obese mice. (4) Reduction of levels of heme through the pharmacological induction of HO-1 reduces the severity of steatosis, inflammation, and fibrosis through the improvement of hepatic mitochondrial function. We and others have shown that heme levels are elevated in lipid laden, unhealthy, terminal differentiated adipocyte, and increase of HO-1; i.e., increase of heme degradation decreases adiposity [38, 39]. Diminishing HO-1 levels are seen in maturing inflamed adipocyte in hepatic tissues [35, 39, 40].

Increased levels of HO-1 shown to play a critical role in the amelioration of oxidative stress, and, in both humans and mice, low levels of HO-1 lead to organ damage [16, 41]. Moreover, overexpression of HO-1 lowers levels of the inflammatory mediators TNF-*α* and IL-6 in the liver of mice [42]. A decrease in HO activity exacerbates mitochondrial lipid peroxidation and mitochondrial dysfunction, while induction of HO-1 upregulates mitochondrial transcription factor [21], all of which support the hypothesis that a reduction in HO activity results in mitochondrial dysfunction and increased insulin resistance [22, 24]. Mitochondrial dysfunction leads to a decrease in beta oxidation in the liver which allows fat to accumulate resulting in a “fatty liver” [43, 44].

The potential beneficial role of decreasing NOV in obesity and metabolic syndrome has been recently described [22]. Increased levels of NOV appear to be a key component of the inflammatory and fibrotic response in the liver of obese mice, and adiposity mediated increase of NOV appears to be involved in hepatic IR and in the pathophysiology of the inflammation and resulting fibrosis. In NOV^−/−^ obese mice, there is a reduction in body weight, a decrease in expression of proinflammatory cytokines and chemokines, and increase in the levels of PGC-1*α* and UCP1. In our obese mouse model, increased levels of HO-1 led to a concomitant reduction in NOV mRNA, as inflammation and markers of fibrosis to the levels of lean animals. This effect was reversed by inhibiting HO activity, not HO-1 protein, by SnMP, a well-known effect for SnMP [37] indicating the pivotal role of HO-1 and HO activity in the regulation of obesity and metabolic syndrome. We speculate that a crosstalk exists between adipose dysfunction and the development of fibrosis and NASH.

Mitochondrial dysfunction is also a key player in the generation of ROS [45], which results in abnormal respiration [43]. Oxidative stress in NAFLD/NASH is associated with the reduced expression of PGC-1*α* in adipose tissue, negatively affecting mitochondrial biogenesis, thereby resulting in the mitochondrial dysfunction that is seen in the development of IR [45]. PGC-1*α* targets SIRT3, a mitochondrial deacetylase, which promotes mitochondrial biogenesis, suppression of ROS [46], and mitochondrial FA oxidation [47]. PGC-1*α* adipocyte knockout mice develop IR and glucose intolerance and consequently elevated levels of circulating lipids and cholesterol [48]. This demonstrates the existence of PGC-1*α* crosstalk between adipocytes and the liver, thereby correlating adipocyte mediated release of inflammatory cytokines with hepatic insulin resistance and steatosis. With HO-1 induction, levels of PGC-1*α* and markers of mitochondrial fusion increase in adipose tissue, oxidative stress decreases, and lipogenesis and liver function in obese mice improve.

A HFD increases the expression of the FIS1 gene, which regulates mitochondrial fission, while concomitantly reducing the expression of those genes responsible for mitochondrial quality control and fusion processes, fueling ROS generation, and causing tissue inflammation [49]. An inverse relationship exists between Mfn2 mRNA levels in skeletal muscles and BMI. Consistent with these observations, skeletal muscle from obese subjects presents an altered, fragmented mitochondrial network, associated with nutrient oxidation, respiratory chain defects, and IR [50]. Additionally, liver MFN2 levels are decreased in obesity, but increased by increasing HO-1 levels, thereby reducing the severity of NASH. In particular, increased levels of HO-1 decrease steatosis and abolish fibrosis, and the NAS score is reduced. In agreement with our data, NASH and fatty liver are both associated with IR, but NASH alone is associated with mitochondrial structural defects [51]. Finally, since hepcidin is released from adipose tissue and is upregulated by NOV, it follows that there would be increased hepcidin in individuals with higher BMI and metabolic abnormalities who would be more likely to develop NASH [1]. Thus, considering only a small percentage of patients with NAFLD progress to NASH, an increase in hepcidin might explain the conversion and associated mitochondrial dysfunction and inflammation. Additionally, differences may exist in patients with fatty liver based on different genetic backgrounds of these individuals (increased heme-NOV and a decrease in HO-1 expression).

**Figure 8:**
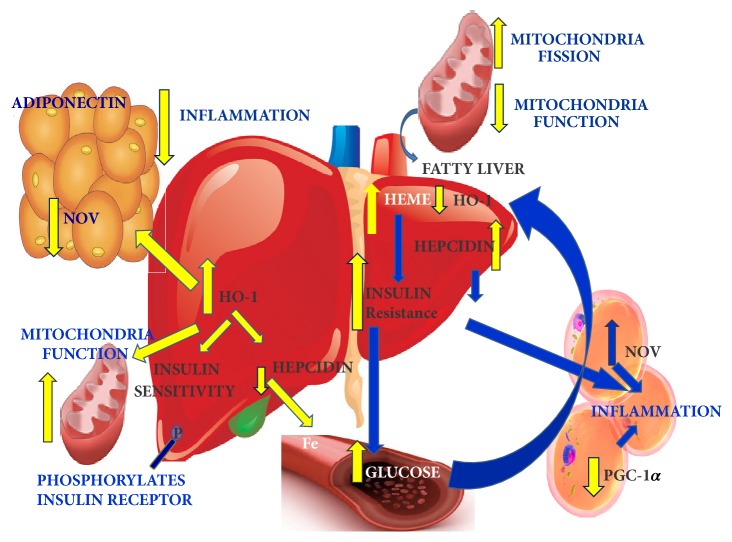
Schematic description of the HO-1 mediated induction of adipose tissue of HF fed mice. As seen in right side panel, decreased HO-1 resulted in an increase of hepatic heme, hepcidin, which is associated with the development and progression of obesity-induced NASH, fibrosis via mitochondrial dysfunction (fission and decreases in COX 2 and 4). Fat expansion was associated with remodeling marked by an increase in adipocyte hyperplasia and hypertrophy leading to proinflammatory molecule NOV, which is associated with a decrease of PGC-1*α* and insulin receptor phosphorylation and eventually development of fibrosis and NAS. In the left panel, induction of HO-1 reduced the severity of steatosis, inflammation, and fibrosis through decrease in NOV and an increase of adiponectin that resulted in the improvement of hepatic mitochondrial integrity, pAMPK-pAKT, and insulin receptor phosphorylation that all in concert leads to an improvement in hepatic function and steatosis.

In conclusion, these data identify that a decrease in adipose and hepatic HO-1 is associated with an increase in adipose-derived NOV activation and that these perturbations can be regarded as key mediators in the development and progression of obesity-induced fibrosis and NASH. Decreased heme levels result in improved mitochondrial function and decreased FAS with an overall reduction in NOV-inflammation, fibrosis, and NASH scores (summarized in Figure 8). As the pursuit of a reliable surrogate marker of inflammation and fibrosis continues, liver histopathology is reflected by the NAFLD Activity Score, which remains the gold standard end-point of therapeutic efficacy. However, future pharmacologic targeting of the NOV/HO-1 axis may prove fruitful in reducing the severity of a disease process that is increasing significantly in prevalence.

## Figures and Tables

**Table 1:** **Histopathological NAS score evaluation.** Degree of steatosis and NASH (grade 0 ≤ 5%; 1 = 5–33%; grade 2 = 34%–66%; grade 3 ≥ 66%), lobular inflammation (0: no foci, 1 < 2 foci per 200x field, 2: 2 to 4 foci per 200x field, and 3: foci per 200x field), hepatocyte ballooning (0: none; 1: rare or few; 2: many), and fibrosis (0: no fibrosis, 1: perisinusoidal or periportal fibrosis, 2: perisinusoidal and portal/perioral fibrosis, 3: bridging fibrosis, and 4: cirrhosis).

NAS pathological score factors	Control	HF	HF + CoPP	HF + CoPP + SnMP
Steatosis	0 (0.67%)	2 (42.24%)	1 (10.28%)	2 (33.05%)
Inflammation	0 (no foci)	2 (2-4 foci/field)	1 (<2 foci/field)	2 (2-4 foci/field)
Ballooning	1 (rare)	2 (many)	1 (rare)	2 (many)
Fibrosis	0 (no fibrosis)	3 (bridging fibrosis)	0 (no fibrosis)	2 (perisinusoidal and portal fibrosis)
NAS value	1	9 NASH	3	8 NASH

## Data Availability

In order to maximize utility of the study results, all data is available to other researchers on request.
